# Alcohol Use Disorder Admissions During the COVID-19 Pandemic: Findings From a Tertiary Community Hospital

**DOI:** 10.7759/cureus.29711

**Published:** 2022-09-28

**Authors:** Richa Tikaria, Maham A Khan, Ling Wang, Adesuwa Olomu, Supratik Rayamajhi, Nishraj Basnet

**Affiliations:** 1 Internal Medicine, Michigan State University, East Lansing, USA; 2 Internal Medicine, Sparrow Hospital, Lansing, USA

**Keywords:** covid-19 retro, hospital admissions, alcohol use disorder, substance abuse, mental health, covid 19 pandemic

## Abstract

Objectives: The first case of Coronavirus disease-19 (COVID-19) in the United States was confirmed by the Centers for Disease Control (CDC) in January 2020. The presence of COVID-19 and the subsequent spread of this disease led to stress, anxiety, grief, and worry. We aimed to study the rate of hospital admission for alcohol use disorder (AUD) before and during the COVID-19 pandemic in a tertiary community hospital in Michigan.

Methods: Two subsets of hospital data were collected for comparison between hospitalized patients before and during the pandemic in a tertiary community hospital. Logistic regression was used to identify the odds ratio of AUD admission rates among all patients in 2020 compared with 2019 while controlling for covariates.

Results: Our data showed a statistically significant increase in AUD patients in 2020 compared to 2019 (3.26% versus 2.50%, adjusted OR=1.44 with P=0.002). In addition, females had significantly lower chances of admission for AUD compared with males (OR=0.22 with P<0.001) and African Americans had significantly lower chances of admission for AUD compared to Whites (OR=0.44 with P <0.001). Divorced patients had a higher probability of admission for AUD compared to married patients (OR=2.62 with P<0.001).

Conclusion: Our study found a significantly higher rate of AUD admissions in 2020 during the COVID-19 Pandemic compared to 2019. Gender, race, age, and marital status are significant risk factors related to AUD admissions.

## Introduction

Pandemics are not just a medical phenomenon; they affect individuals and society on many levels, causing disruptions. Panic and stress have also been linked to outbreaks [1]. Several studies have identified an increase in mental health problems, alcohol, and substance abuse during the ongoing COVID-19 (Coronavirus disease-19) pandemic [2-4]. This increase was most likely secondary to stress due to a combination of working from home, temporary unemployment for others, home-schooling of children, lack of physical contact, and unpredictability associated with the pandemic. Stress is a prominent risk factor for the onset and maintenance of alcohol misuse. For example, chronic alcohol use results in neuroadaptations in stress and reward pathways, which lead to dysfunctional hypothalamic-pituitary-adrenocortical and sympathetic adrenomedullary axes, characterized by dysregulation of the cortisol response and deficits in emotional regulation [5]. According to the 2019 National Survey on Drug Use and Health conducted in the United States of America, nearly 15 million people ages 12 and older had an alcohol use disorder (AUD) [6]. An AUD is defined as a chronic brain disorder marked by compulsive drinking, loss of control over alcohol use, and negative emotions when not drinking. Of the 15 million people with AUD, 6.8% were men and 3.9% were women [7]. This prevalence of alcohol abuse and dependence translates into about 18.5% of emergency department visits on average [8]. One of the studies reported an increase in the frequency of alcohol consumption in 2020. On average, alcohol was consumed one day more per month by three out of four adults. For women, there was also a significant increase of 0.18 days of heavy drinking, from a 2019 baseline of 0.44 days, which represents an increase of 41% over the baseline [9]. In 2020, alcohol use will increase worldwide. During the first wave of COVID-19 in the USA, brick-and-mortar and online sales of alcohol skyrocketed [10]. We aimed to study whether there was a concomitant increase in inpatient admissions for alcohol use disorder in various subsets of the patient population during the pandemic.

## Materials and methods

The study was approved by the Michigan State University Biomedical and Health Institutional Review Board. The study was reviewed by the Institutional Review Board (IRB) through the Non-Committee Review procedure. The IRB has found that this study protects the rights and welfare of human subjects and meets the requirements of MSU's Federal Wide Assurance (FWA00004556) and the federal regulations for the protection of human subjects in research (e.g., 2018 45 CFR 46, 21 CFR 50, 56, and other applicable regulations).

We obtained admission and demographic data for all adult hospitalized patients (age 18 or above at admission) at the local tertiary level hospital dating between March 1, 2019 to December 31, 2019, and March 1, 2020 to December 31, 2020. Two subsets of hospital data were collected for comparison between hospitalized patients before and during the pandemic. Patients with an F10 principal or admission diagnosis, i.e., an alcohol-related disorder from the International Classification of Diseases Tenth Revision (ICD-10), were included in the study cohorts. To incorporate the most recent discharge diagnoses, data were extrapolated from the discharge summaries. Data were stratified on subsets of age, gender, employment, race, and marital status. Statistical testing was done using Chi-square tests to compare gender distribution in the same months in 2019 and 2020. Significance was set at two-tailed p < .05. All calculations were done using SAS statistical software version 9.4 (SAS Institute Inc., Cary, NC). The probability of admission was calculated by logistic regression. Data analysis was performed from March to December. These specific monthly data were chosen to curb seasonal bias and were coincident with the first statewide lockdown of the COVID-19 pandemic.

## Results

There were a total of 15,625 patients admitted from March 1, 2019 to December 31, 2019, and 13,854 from March 1, 2020 to December 31, 2020, to Sparrow Hospital. Out of these, 391 admissions were attributed to alcohol use disorder (overall mean [SD] age 46.7 [12.8]; 87 [22.3%] women; 314 [80.3%] White patients; 40 [10.2%] African American patients; 20 [5.1%] Hispanic patients; 238 [60.9%] unemployed patients; 56 [14.3%] married) in 2019. In 2020, a total of 451 patients (overall mean [SD] age 44.4 [12.6]; 119 [26.4%] women; 366 [81.2%] White patients; 45 [10%] African American patients; 24 [5.3%] Hispanic patients; 299 [66.3%] unemployed patients; 78 [17.3%] married) were admitted secondary to AUD (Table 1).

**Table 1:** Characteristics of patients admitted with alcohol use disorder in 2019 and 2020.

Age at admission (years)		2019	2020	p-values
		Mean (SD)	Mean (SD)	
		46.7(12.8)	44.4(12.6)	0.01
Variable	Category	N (%)	N (%)	
Age groups	18-39	132(41.1%)	183(46.3%)	0.16
	40-60	189(58.9%)	212(53.67%)	
Sex	Female	87(22.3)	119(26.4)	0.16
	Male	304(77.8)	332(73.6)	
Employment status	Missing	2(0.6)	6(1.4)	0.06
	Full time	58(14.8)	68(15.1)	
	Not employed	238(60.9)	299(66.3)	
	Part-time	14(3.6)	18(4)	
	Retired	62(15.9)	38(8.4)	
	Self-employed	16(4.1)	18(4)	
	Student - full time	1(0.3)	4(0.9)	
Race	Black or African American	40(10.2)	45(10)	0.94
	Hispanic	20(5.1)	24(5.3)	
	Other	17(4.4)	16(3.6)	
	White	314(80.3)	366(81.2)	
Marital status	Missing	3(0.8)	2(0.4)	0.05
	Divorced	95(24.3)	88(19.5)	
	Married	56(14.3)	78(17.3)	
	Separated	6(1.5)	19(4.2)	
	Significant other	3(0.8)	8(1.8)	
	Single	205(52.4)	239(53)	
	Widowed	23(5.9)	17(3.8)	

**Figure 1:**
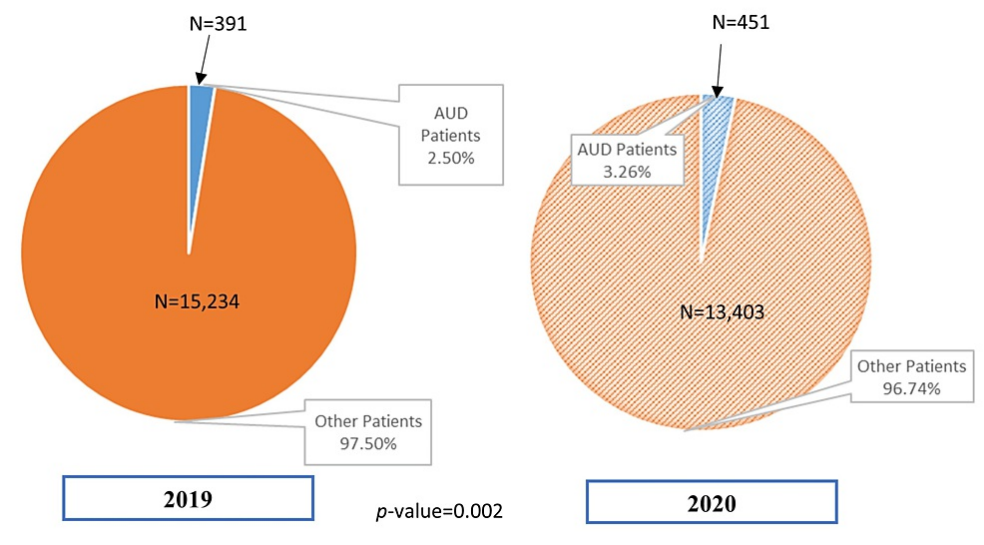
Percent of patients admitted with alcohol use disorder in 2019 and 2020.

**Figure 2:**
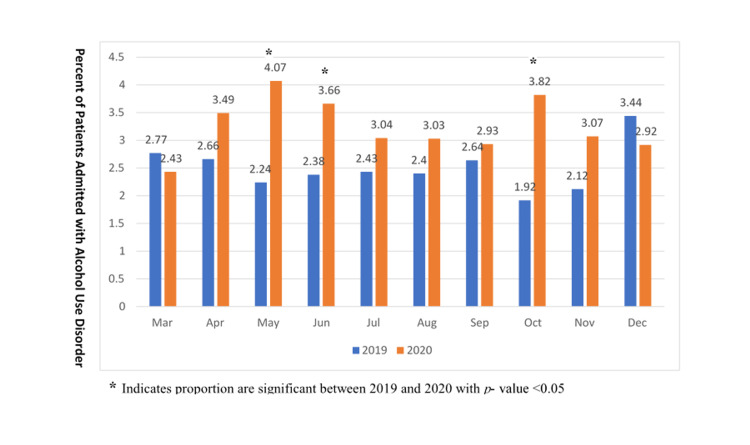
Monthly percent of patients admitted for alcohol use disorder in 2019 and 2020.

Our data showed a statistically significant increase in the number of admissions for alcohol use disorder patients, with a p-value of 0.002. In 2019, 391/15,625 (2.50%) patients were admitted for AUD compared to 451/13854 (3.26%) in 2020 (Figure 1). The increase was statistically significant in the months of May, June, and October, with p- values <0.05 (Figure 2). Our data also showed that patients admitted for AUD were younger in age in 2020 (mean age of 44.4 with SD 12.6) compared to 2019 (mean age of 46.7 with SD 12.8) with a p-value of 0.01. We compared the patients to two subsets of ages: 18-39 years old and 40-60 years old. The number of patients aged 18-39 years increased in 2020 (46.3%) compared to 2019 (41.1%). The percentage of African American patients admitted in 2019 was 10.2%, similar to 10% in 2020. Similarly, 80.3% of white AUD patients were admitted in 2019, and 81.2% in 2020. More married patients were admitted in 2020 (17.3%) compared to 2019 (14.3%) with a p-value of 0.05. (Table 1).

It was found that after adjusting for covariates such as gender, race, marital status, age, and employment status, patients had a significantly higher probability of being admitted with AUD in 2020 compared to 2019 (OR=1.44 with P-value<0.0001). Younger patients (18-39 years old) had a significantly higher chance of being admitted with AUD (OR=1.33 with P-value=0.001) compared with patients 40-60 years old. Unemployed patients had a significantly higher chance of being admitted with AUD (OR=1.93 with P-value<0.001) compared with employed patients. Female patients had a significantly lower probability of being admitted with AUD (OR=0.22 with P-value<0.0001) compared with male patients. Compared with White, African Americans and other races had significantly lower chances of being admitted with AUD (OR=0.44 and 0.56 with P-values<0.0001 and 0.007, respectively). Compared with married patients, patients who were divorced, separated, or single had a significantly higher chance of being admitted with AUD (OR=2.62, 2.11, and 1.90 with P-values<0.0001, = 0.002, and <0.0001, respectively) (Table 2).

**Table 2:** Logistic regression results predicting probability of admitting with alcohol use disorder 2019-2020.

Variables	Odds ratio	95% Wald		P-values
		Confidence limits		
2020 vs 2019	1.437	1.231	1.677	<0.0001
Female vs male	0.22	0.184	0.263	<0.0001
Black or African American vs White	0.441	0.337	0.576	<0.0001
Hispanic vs White	1.224	0.882	1.698	0.2277
Other vs White	0.563	0.37	0.857	0.0074
Divorced vs married	2.623	2.024	3.4	<0.0001
Separated vs married	2.113	1.316	3.391	0.002
Significant other vs married	1.55	0.792	3.033	0.2006
Single vs married	1.904	1.515	2.392	<0.0001
Widowed vs married	2.537	1.518	4.241	0.0004
Age: 18-39 vs 40-60	1.331	1.122	1.579	0.001
Unemployed vs employed	1.926	1.622	2.286	<0.0001

We tabulated comparison of unemployed patients admitted in 2019 to unemployed patients admitted in 2020 for alcohol use disorder (Table 3). We observed that the unemployed patients (not including employed, students, and retired) who were admitted for AUD during the pandemic formed a larger fraction of the patients compared to the year prior (60.87% in 2019 versus 66.30% in 2020). In particular, in the month of October, significantly more unemployed patients were admitted for AUD in 2020 when compared to 2019. (72.4% versus 48.3% with a p-value of 0.02).

**Table 3:** Proportion of female patients/unemployed patients admitted for alcohol use disorder by months in 2019 and 2020.

Months	2019		2020		p-Values	2019		2020		p-Values
	Number of female patients	%	Number of female patients	%		Number of unemployed patients	%	Number of unemployed patients	%	
											Mar	8	18.6	9	30	0.26	21	48.84	21	70	0.07
Apr	5	11.9	12	34.29	0.02	29	69.05	22	62.86	0.57
May	8	22.22	12	24	0.85	21	58.33	30	60	0.88
Jun	7	19.44	9	16.98	0.77	20	55.56	37	69.81	0.17
Jul	7	17.95	11	25	0.44	24	61.54	31	70.45	0.39
Aug	8	21.05	14	32.56	0.25	24	63.16	24	55.81	0.50
Sep	13	31.71	8	17.39	0.12	28	68.29	32	69.57	0.90
Oct	9	31.03	18	31.03	1.00	14	48.28	42	72.41	0.03
Nov	4	12.5	12	25.53	0.16	20	62.5	29	61.7	0.94
Dec	18	32.73	14	31.11	0.86	37	67.27	31	68.89	0.86
Total	87	22.25	119	26.38	0.19	238	60.87	299	66.30	0.12

Table 3 also tabulated a comparison of female patients admitted in 2019 to female patients admitted in 2020 for alcohol use disorder. While females admitted during April 2019 for alcohol use disorder comprised 11.9% of the total number of patients admitted for AUD, this increased to 34.3% in April 2020. In April, it was found that there were significantly more female patients admitted for AUD from 2019 to 2020 (p-value=0.02). (Table 3). There was no statistically significant difference in admissions of female patients for the duration of the measurement period (March-December).

## Discussion

We found a statistically significant increase in the number of admissions for alcohol use disorder during the first wave of the pandemic (2020) in the USA, compared to the year prior. In 2019, 2.50% of patients were admitted for AUD, compared to 3.26% in 2020 (Figure 1). The increase was statistically significant in the months of May, June, and October (p-values < 0.05; Figure 2).

Our study also found a statistically significant difference in the percentage of AUD females admitted to the hospital in the month of April compared to the year prior (Table 3). Women have also been subject to increased abuse and loss of jobs, potentially worsening the existing gender disparity and further exacerbating mental health problems in this demographic [11]. Unemployment was another major contributor to alcohol-related admissions during the pandemic. The expected number of job losses due to COVID-19 was taken from the International Labor Organization's press release dated March 18, 2020, reporting a decline of 24.7 million jobs as a high scenario and 5.3 million jobs lost as a low scenario [12]. Our study showed the most significant effect in the month of October, when the impact of unemployment was high. According to the Current Population Survey in the Detroit Metro area, the unemployment rate was highest in March-April (around 45%) but improved to less than 20% in August. There was another upward trend in September-October and the unemployment rate started to go above 20%. [13] At the start of this pandemic, several state governments imposed stay-at-home orders, requiring schools, restaurants, and non-essential businesses to close to mitigate the spread of the disease. During this period, alcohol consumption increased considerably. There was a 54% surge in national alcohol sales during the first week of the pandemic, and subsequent reports indicated persistent increases in rates of alcohol consumption [14]. The rate of alcohol abuse increased more significantly in women compared to men secondary to job losses disproportionately impacting women [15] and with many women playing multiple, conflicting roles of at-home worker, primary caregiver, and homeschooling teacher [16].

The pandemic has both short- and long-term implications for mental health and substance use, particularly for groups at risk of new or exacerbated mental health disorders and those facing barriers to accessing care. A common result of long-term alcohol use is the development or exacerbation of depression. Several studies have shown an increase in suicide rates secondary to increased anxiety, depression, alcohol, and other substance use disorders [17].

Limitations

There are limitations to our study. We had a small sample size and our sample frame was limited to one tertiary community hospital during the lockdown period, limiting the generalizability of our results. We did not follow up with patients to see how many had readmissions for relapses with alcohol abuse. However, our findings highlighted the impact of the COVID-19 pandemic on the mental health of the general population, using alcohol as a coping mechanism during times of stress.

## Conclusions

Our results highlight the impact of a pandemic on alcohol abuse and subsequent hospital admissions for alcohol use disorder. Our study found a higher rate of AUD admissions in 2020 during the COVID-19 pandemic compared to 2019. Gender, race, age, and marital status are significant risk factors related to AUD admissions. Women have been subject to increased abuse and loss of jobs, potentially worsening the existing gender disparity and further exacerbating mental health problems in this demographic. Unemployment was another major contributor to alcohol-related admissions during the pandemic. This calls for attention from public health authorities and healthcare practitioners to anticipate and ensure support for subgroups of patients coping with the stressors of a pandemic.
